# Bimanual finger coordination in professional and amateur darbuka players

**DOI:** 10.1007/s00221-023-06703-9

**Published:** 2023-09-26

**Authors:** Kazuaki Honda, Shinya Fujii

**Affiliations:** 1 Graduate School of Media and Governance, Keio University, 5322 Endo, Fujisawa, Kanagawa 252-0882 Japan; 2 NTT Communication Science Laboratories, NTT Corporation, 3-1 Morinosato Wakamiya, Atsugi, Kanagawa 243-0124 Japan; 3 Faculty of Environment and Information Studies, Keio University, 5322 Endo, Fujisawa, Kanagawa 252-0882 Japan

**Keywords:** Motor skills, Tapping, Darbuka players, Coordination, Cross-recurrence quantification analysis

## Abstract

Professional hand percussionists who play the darbuka (a drum from the Middle East) show fast and stable bimanual finger coordination compared to amateur players. A cross-recurrence quantification analysis clarifies how stable bimanual coordination is achieved by dissociating stochastic noise and attractor strength in the dynamic system. This study employed a cross-recurrence quantification analysis to examine professional and amateur darbuka players’ fast and stable bimanual finger coordination. Eight professional and eight amateur percussion players participated in the study and played a darbuka with their right and left ring fingers, alternating as fast as possible for 12 s. We then analyzed the finger position data and calculated the stochastic noise and attractor strength from the density and the longest diagonal line in the recurrence plot, respectively. We used linear mixed-effects models to test whether stochastic noise and attractor strength differed between professional and amateur players. The results indicate that professional darbuka players achieve fast and stable bimanual finger coordination by enhancing attractor strength rather than reducing stochastic noise in the dynamic system.

**Supplementary Information:**

The online version contains supplementary material available at 10.1007/s00221-023-06703-9.

## Introduction

Musicians exhibit skilled bimanual coordination. Illustrative examples include a pianist’s fingers gracefully sweeping across the keyboard to produce complex harmonies, and a percussionist’s hands striking the drums rhythmically, each beat precisely timed. These are not the only demonstrations of human motor coordination; skilled bimanual coordination is also evident in everyday tasks such as typing on a keyboard. A multitude of studies on human motor learning have evidenced that motor performance tends to become less variable with practice (Müller and Sternad 2009). With fast rhythmic tapping movements, motor variability is measured through inter-tap interval (ITI) variability (Peters 1976; Schmidt et al. 2000; Aoki and Kinoshita 2001; Sommervoll et al. 2011). ITI variability usually increases with increased tapping speed, producing a trade-off between variability and speed in bimanual coordination (Sommervoll et al. 2011).

We increase speed (while increasing accuracy) and reduce variability with practice, and this indicates motor learning. For example, finger-tapping movements become faster but less variable after prolonged practice (Peters 1976). This study’s central question investigates how less variable (stable) but fast movements emerge during skilled bimanual coordination. Musicians are an ideal population for investigating how human motor systems adapt to acquire bimanual coordination skill (Münte et al. 2002; Zatorre 2005; Schlaug 2015). Many finger-tapping studies have reported that musicians show less ITI variability than unskilled controls (Yamanishi et al. 1980; Jäncke et al. 1997; Verheul and Geuze 2004; Aoki et al. 2005; Fujii and Oda 2006; Fujii et al. 2010; Madison et al. 2013; Honda and Fujii 2022). Skilled pianists and drummers exhibit stable bimanual coordination (Yamanishi et al. 1980; Fujii et al. 2010). Importantly, both stable and fast manual movements have been observed in skilled musicians compared to unskilled controls such as non-musicians and amateur musicians (Jäncke et al. 1997; Aoki et al. 2005; Fujii and Oda 2006; Honda and Fujii 2022). For example, we recently reported that professional hand percussionists who play the darbuka (Karaol and Doğrusöz 2014) can tap faster than amateur players (Honda and Fujii 2022). However, it is unclear how skilled darbuka players accomplish stable and fast bimanual movements.

To understand how stable coordination patterns emerge in the human motor system, theoretical studies have proposed dynamical system models of bimanual coordination using coupled oscillators (Yamanishi et al. 1980; Haken et al. 1985; Schöner et al. 1986). Kelso (1984) reported that bimanual coordination becomes unstable, and a phase transition from anti-phase to in-phase occurs as movement frequency increases. Haken et al. (1985) modeled the phenomenon as a motion equation, in which coordination stability was depicted as the relative phase $\phi = ({\theta }_{r }-{\theta }_{l})$, the phase-angle difference between the right and left hands (see Eq. 1). When the relative phase is in-phase ($\phi =0^\circ$), homologous muscles in both hands are synchronously contracted. When the relative phase is anti-phase ($\phi =180^\circ )$, homologous muscles of both hands are alternately contracted. The phase transition phenomenon can be described as follows:

1 $$\dot{\phi} = \Delta \omega - a \sin \phi - 2b\,\sin \,2\phi + \sqrt Q \xi_{t} ,$$

where $\dot{\phi }$ denotes the time derivative of the relative phase. The $a$ and $b$ parameters denote the coupled oscillators’ strengths; ${\xi }_{t}$ is a Gaussian white noise process with a stochastic force having strength $Q$ (Schöner et al. 1986); $\Delta \omega$ is a detuning parameter that corresponds to the eigen frequency difference between the coupled oscillators (Kelso et al. 1990). In Eq. 1, the system has two fixed points or attractors at $\phi =0^\circ$ or $180^\circ$. The attractor strength depends on the magnitudes of $a$, $b$, and $\Delta \omega$. Increased movement speed is known to change the ratio of $b/a$*,* leading to unstable attractor strength at $\phi =180^\circ$. Increased movement speed is also known to increase the noise process with stochastic force $Q$ (Pellecchia et al. 2005; Shockley and Turvey 2005).

Two possible mechanisms underlie stable and fast bimanual coordination based on a dynamical system model: stabilizing the attractor strength and decreasing the stochastic noise (Schöner et al. 1986). It is possible that professional musicians simply show lower noise magnitude. On the other hand, Yamanishi et al. (1980) found that skilled pianists’ lower variability resulted from a weak interaction between two coupled rhythmic oscillators. In addition, professional drummers performed stable fast tapping because of lower asymmetry causing small $\Delta \omega$ (Fujii et al. 2010). These findings suggest that professional musicians have more stable attractors for fast bimanual coordination. However, existing studies have not directly evaluated stochastic noise and attractor strength differences between skilled and non-skilled musicians.

Recurrence quantification analysis (RQA) is an appropriate method for understanding this difference (Eckmann et al. 1987; Zbilut and Webber 1992; Webber and Zbilut 2005). RQA analyzes a dynamical system’s structure by embedding the dynamics in a higher dimensional phase space. If state ${x}_{i}$ is included in the neighborhood of another state ${x}_{j}$, then ${x}_{i}$ and ${x}_{j}$ are treated as recurrent states. RQA is often expanded to cross-recurrence quantification analysis (CRQA), which examines interlimb coordination (Pellecchia et al. 2005; Richardson et al. 2007). To analyze the two time series’ (right and left hand movements), each time series is embedded in the same phase space. If the state of ${x}_{i}$ is included in the neighborhood of ${y}_{j}$, then ${x}_{i}$ and ${y}_{j}$ are said to be cross-recurrence points. Recurrence (or cross-recurrence) states are plotted on a two-dimensional (*N* × *N*) array, where dots mark recurrences and both axes (N in length) represent the location in time along the trajectory. Two metrics can be calculated from the recurrence plot: %REC and Lmax. %REC is “the percentage of recurrent points falling within the specified radius”, and Lmax is “the length of longest diagonal line in recurrence plot” (Webber and Zbilut 2005). Richardson et al. (2007) showed that these two indices independently measure the relative effects of stochastic noise $Q$ and attractor strength on movement variability. Their results showed that a higher %REC indicated lower stochastic noise magnitude, and a longer Lmax indicated a more stable attractor strength. Thus, CRQA clarifies differences between skilled and non-skilled musician’s fast bimanual coordination.

This study employed CRQA to investigate whether stochastic noise and attractor strength differ between professional and amateur darbuka players in a bimanual finger-tapping task. Jäncke et al. (2000) found that professional musicians required fewer active neurons to perform given finger movements. Thus, we assumed that professional musicians have lower signal-dependent noise. Studies have also shown that learning increases attractor strength and reduces movement variability (Zanone and Kelso 1992, 1997). Fujii et al. (2010) showed that smaller $\Delta \omega$ in professional drummers was associated with greater attractor strength. These studies suggest that professional darbuka players may have increased attractor stability.

## Methods

### Participants

Sixteen Japanese darbuka players (all male) participated in the study; eight professional players (mean age = 37.25 years, standard deviation [SD] = 5.66, range = 30–50 years) and eight amateur players (mean age = 41.50 years, SD = 9.34, range = 28–55 years). Professional players earned a living through musical performances and instructing students, whereas amateur players did not. Professional players began playing darbuka at an earlier age (mean = 24.38 years, SD = 4.27, range = 21–34 years) than amateur players (mean = 35.63 years, SD = 10.80, range = 25–51 years; *t* (14) = 2.74, *p* = 0.02), and darbuka training duration was significantly longer in professional players (mean = 13.75 years, SD = 2.77, range = 8–17 years) than in amateur players (mean = 6.38 years, SD = 4.92, range = 4–18 years; *t* (14) = − 3.69, *p* = 0.02). We used the Edinburgh Handedness Inventory to determine participants’ handedness (Oldfield 1971). Professional players’ mean laterality quotient was 92.37 (SD = 10.54, range = 80–100) and that of amateur players was 92.37 (SD = 15.01, range = 60–100). Professional and amateur players were matched for sex, age, and handedness. The experimental procedure was approved by the Ethics Committee of the Keio University Shonan Fujisawa Campus (No. 161), and all participants provided informed consent.

### Setup

Drumming chair height and position were adjusted for each participant to ensure comfort. Participants were asked to hold a darbuka (aluminum die-cast model, Egygawhara) under their left upper arm. We placed a camera (FDR-AX700, Sony Corporation) in front of participants, and recorded video at 59.94 Hz. We also recorded sound data; the tapping performances analyzed from the sound data were published in our previous study (for details, see Honda and Fujii (2022)). One professional player’s data were excluded from the analysis because of recording errors.

### Task

**Fig. 1:**
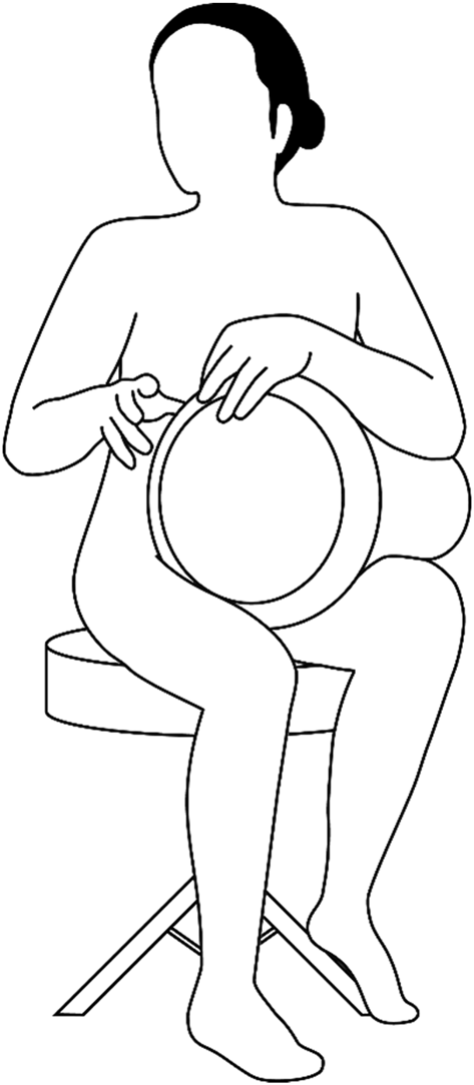
Darbuka player. Players use their right and left ring fingers to play the darbuka

We used a double-finger coordination task (Honda and Fujii 2022) for which participants were asked to coordinate their right and left ring fingers alternately and as fast as possible (Fig. 1). They started tapping from their preferred hand for 12 s after a start call from the experimenter. As we were investigating actual darbuka playing natural performance, participants were asked to tap with their fingers, but were allowed to use upper arm joint movements without any constraints, as they usually play the instrument. Participants performed three trials each with a one-minute rest between trials to prevent fatigue. We decided to conduct three trials based on previous fast tapping studies (Aoki et al. 2005; Fujii and Oda 2006). The right and left fingers were used because darbuka is primarily played using bimanual finger coordination.

### Data analysis

#### Preprocessing

The recorded video data were trimmed to 12-s clips for each trial. DeepLabCut (Mathis et al. 2018) was then used to annotate right and left ring fingers’ positions. After manually annotating the right and left ring fingertips for 20 images in each trial, the network was trained for 500000 iterations using ResNet 50. Finally, we checked the outlier data, and manually fixed and retrained the network for 398000 iterations.

First, we truncated the first and last 1-s data to eliminate the effects of startup and final slowing and used the data during the middle 10-s period. To remove outliers from the annotation data, we used likelihood, computed using DeepLabCut with a threshold of 0.95. Points with likelihood less than threshold were replaced with missing data, and the missing data were incorporated with a piecewise cubic Hermite interpolating polynomial. The fingertips’ trajectory during fast tapping was elliptical. During darbuka play, each ring finger moves to tap a point near the long axis. Therefore, the trajectory was rotated such that the positive direction of the x-axis coincided with the tapping points. To observe coordination during fast movement, we removed the drifts or irrelevant noise caused by the position drifts by applying a 4–9 Hz bandpass filter (4th order Butterworth). The filter cutoff frequency was set to include the Q1–Q3 range of the tapping frequency [6.07–7.64 Hz] calculated from all ITIs shown in our previous study. We used the x-axis position of the rotated trajectory for relative phase analysis and CRQA.

#### Relative phase analysis

To quantify the degree of variability in bimanual coordination, we evaluated the relative phase distribution by calculating the length of the resultant vector (LRV) (see a paper by Berens (2009) for more details about the circular statistics). The LRV was calculated from the synthesized vector of the relative phases between the two time series’ (i.e., right and left ring fingers’ positions), indicating how the relative phases are aligned and related to the standard deviation of the relative phase ($SD\phi$). The LRV is defined as follows:

2 $${\text{LRV = }}\frac{1}{N}\left| {\sum\limits_{t = 1}^{N} {e^{i\pi \phi_t} } } \right|,$$

where ${\phi }_{t}$ is a relative phase at the $t$th frame and $N$ is number of frames. The LRV ranges from 0 to 1; higher values indicate that the participant tapped consistently in a certain relative phase. To calculate the LRV, we used the Hilbert transformation to calculate the instantaneous relative phase between right and left ring fingers’ positions. We also calculated the angle of the resultant vector for each observation.

#### CRQA

We conducted the CRQA using the following process: first, we converted the position data into unit interval ranges to remove scale differences (for more detailed information, see a tutorial by Webber and Zbilut (2005)). Three parameters were used to obtain the CRQA results: time delay $\tau$, embedded dimension $d$, and threshold radius $r$, which were determined using the bimanual coordination method described by Richardson et al. (2007).

Time delay, the temporal offset between copies of the time series, was used to reconstruct the higher dimensional phase space. In stationary periodic or oscillatory systems, a quarter cycle of frequency is appropriate for the time delay. Therefore, we set the time delay of $\tau = 2$ because the average frequencies of the fast bimanual coordination were 7.19 Hz (SD = 1.24) in the professional players and 6.67 Hz (SD = 2.30) in the amateur players. We also calculated the average mutual information (Wallot and Mønster 2018) and confirmed that the optimal value of the time delay was $\tau = 2$ (see also Fig. S1).

The embedding dimension was the number of dimensions used to determine the reconstructed system trajectory. Related studies found that the appropriate number of embeddings to capture the movement dynamics in rhythmic limb movement was five (Mitra et al. 1997; Goodman et al. 2000). Therefore, we used an embedding dimension $d= 5$ for the analysis (see also Fig. S2).

Finally, the last parameter, the threshold radius $r$ was used to determine whether states ${x}_{i}$ and ${y}_{i}$ were recurrent in phase space. If $r$ is too small, only the noise is measured; conversely, an excessively large value of $r$ results in recurrent points that do not reflect the local structure of the focused dynamical system. To determine the value of $r$, we used the following criteria: first, the selected $r$ should result in values of %REC and Lmax that are included in open intervals with floor and ceiling (i.e., %REC ∈ (0, 100) and Lmax ∈ (0, maximum possible line length)). Second, the value of $r$ should be chosen from a region of $r$ values that result in linear scaling of %REC values on a log–log plot. We confirmed that *r* took linear scaling in an interval from 0.09 to 0.21 with a participant’s data. Based on the above criteria, we chose the threshold radius $r$ (0.2) to calculate %REC and Lmax. CRQA was performed using “crqa” R package (Coco and Dale 2014; Wallot and Mønster 2018).

#### Statistics

We then pooled LRV, %REC, and Lmax from the three trials for each participant and created linear mixed-effects models (LMMs) to test hypotheses regarding tapping variability. We first entered group (professional/amateur) as a fixed effect in the LMM to test whether group differences affected tapping variability. Participants and trials were entered as random effects to account for interindividual and intertrial differences. Statistical analyses were performed using R software. The LMMs were performed using the “lmer” function in the “lme4” R package (Bates et al. 2015). Type-3 Wald Chi-Square tests were used to test significant main effects in the LMMs. The results of each statistical analysis were deemed significant at *p* < 0.05. We calculated the partial eta squared (${\eta }_{p}^{2}$) values as the effect sizes. To evaluate the robustness of our data with a small sample size, we used an additional k-fold cross-validation analysis when we obtained a significant main effect (de Rooij and Weeda 2020). We then compared the prediction performance of two regression models: Model 1, with only the intercept, and Model 2, with the intercept and group. We set the number of folds to five because of the small sample size (de Rooij and Weeda 2020), and repeated the validation 2000 times. Each observation was not independent in the LMMs; thus, we applied blocking cross-validation to put all data from the same participant into the same fold (Roberts et al. 2017).

## Results

### Relative phase analysis

**Fig. 2:**
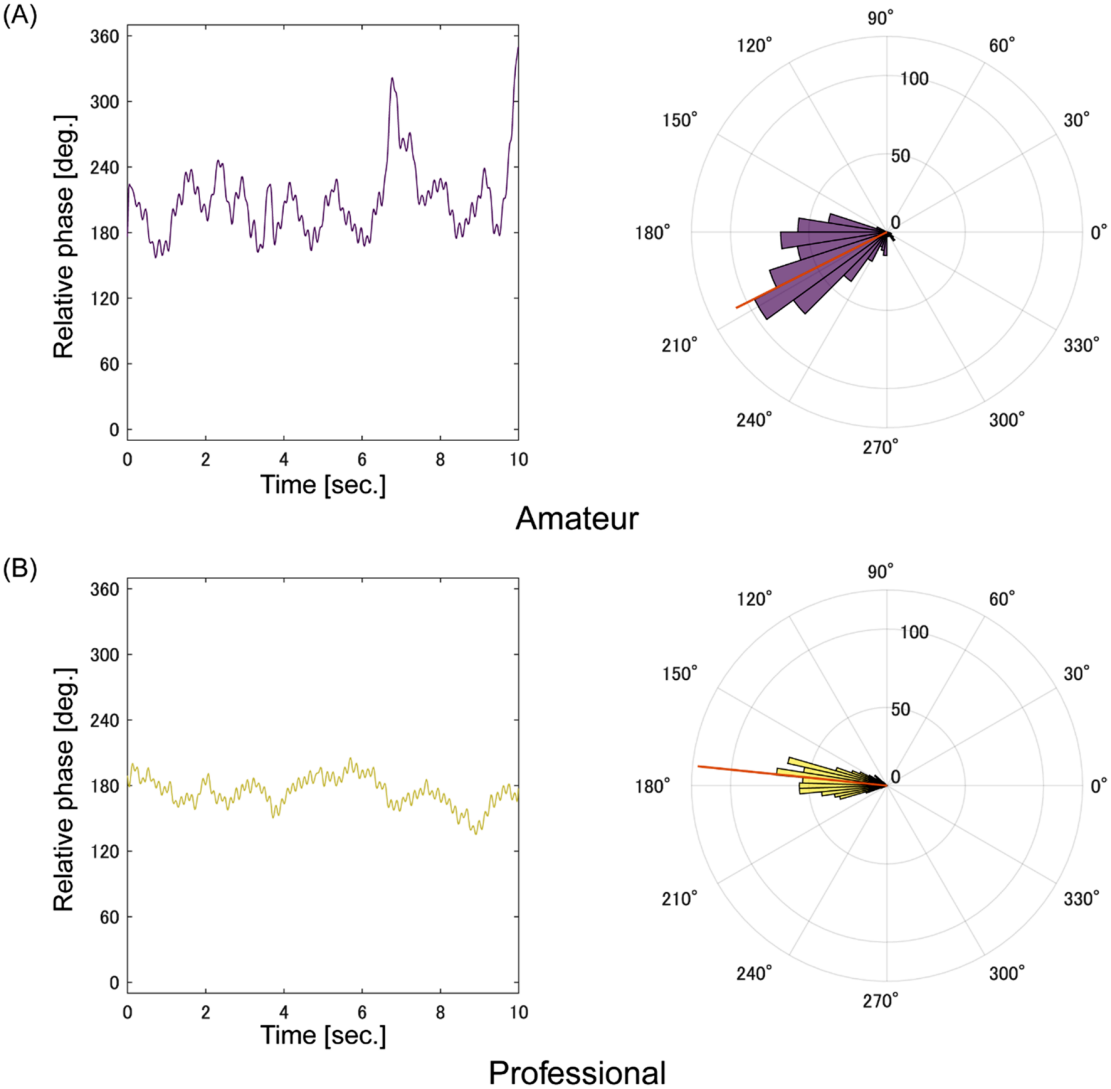
Typical examples of time series and circular histograms of relative phase for **A** an amateur player (purple) and **B** a professional player (yellow). The orange lines in the circular plot represent the resultant vectors

**Fig. 3:**
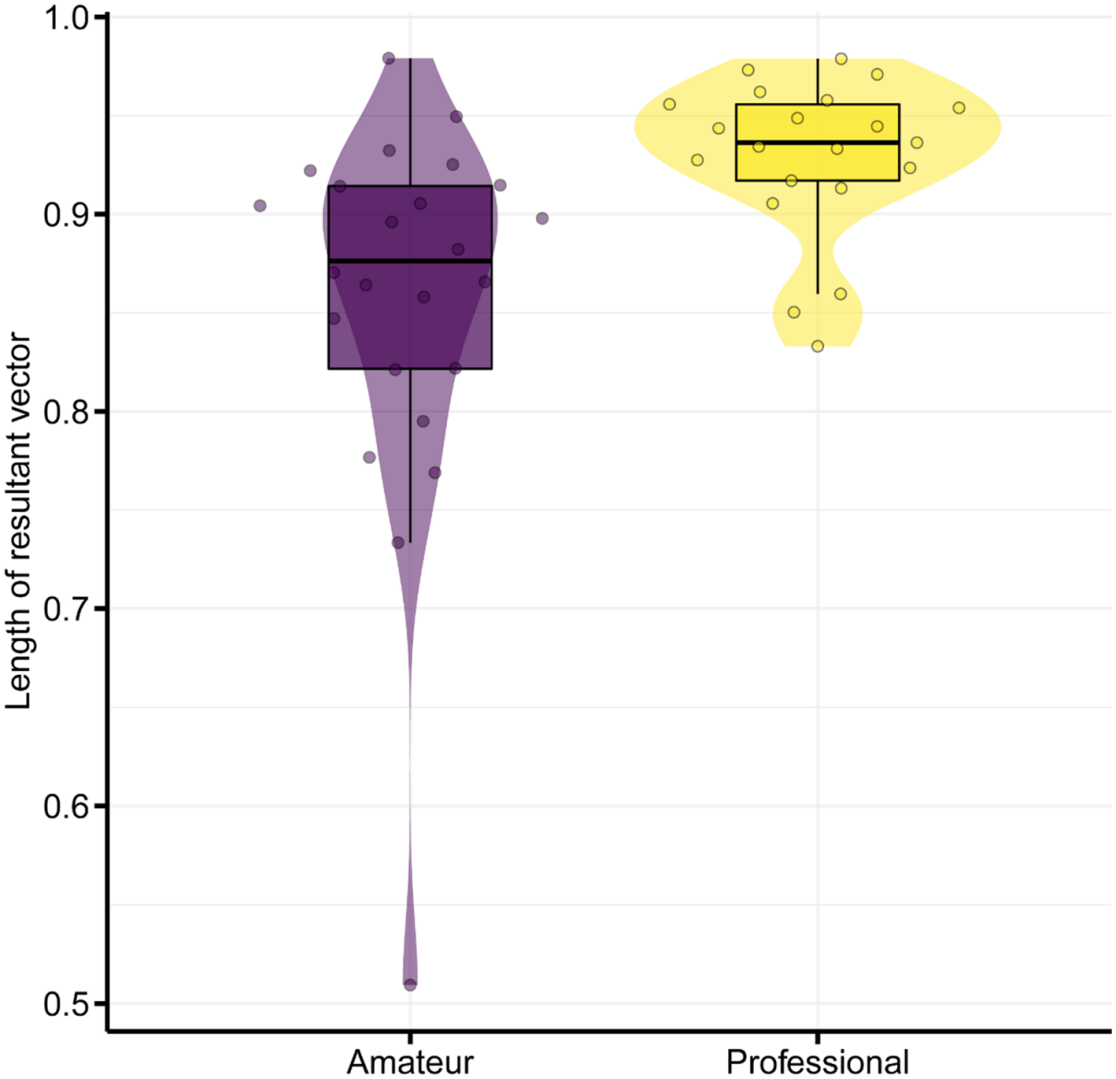
LRV of amateur and professional darbuka players. Each dot represents an observation for a participant’s trial

Figure 2 shows professional and amateur players’ relative phase of bimanual tapping. The mean LRV of bimanual tapping in the amateur players was 0.86 while that in the professional players was 0.93 (Fig. 3). The LMM on the LRV showed that the main effect of group was significant (${\chi }^{2}$(2) = 7.37, *p* < 0.01, ${\eta }_{p}^{2}$ = 0.36); professional players showed less relative phase variability (Fig. 3, *β* = 0.073, *t* = 2.71, *95% CI* 0.021–0.13). The blocking cross-validation results for the relative phase data showed that Model 2 (intercept and group) won in 1984 out of 2000 cases and returned a lower prediction error than Model 1 (intercept only). Additionally, we confirmed that the LMM on the LRV, excluding the outlier value of one amateur darbuka player, consistently showed the significant main effect of group (${\chi }^{2}$(2) = 6.05, *p* = 0.014, ${\eta }_{p}^{2}$ = 0.32). The LMM on the angle of the resultant vector showed that the main effect of group was not significant (${\chi }^{2}$(2) = 0.24, *p* = 0.63).

### CRQA

**Fig. 4:**
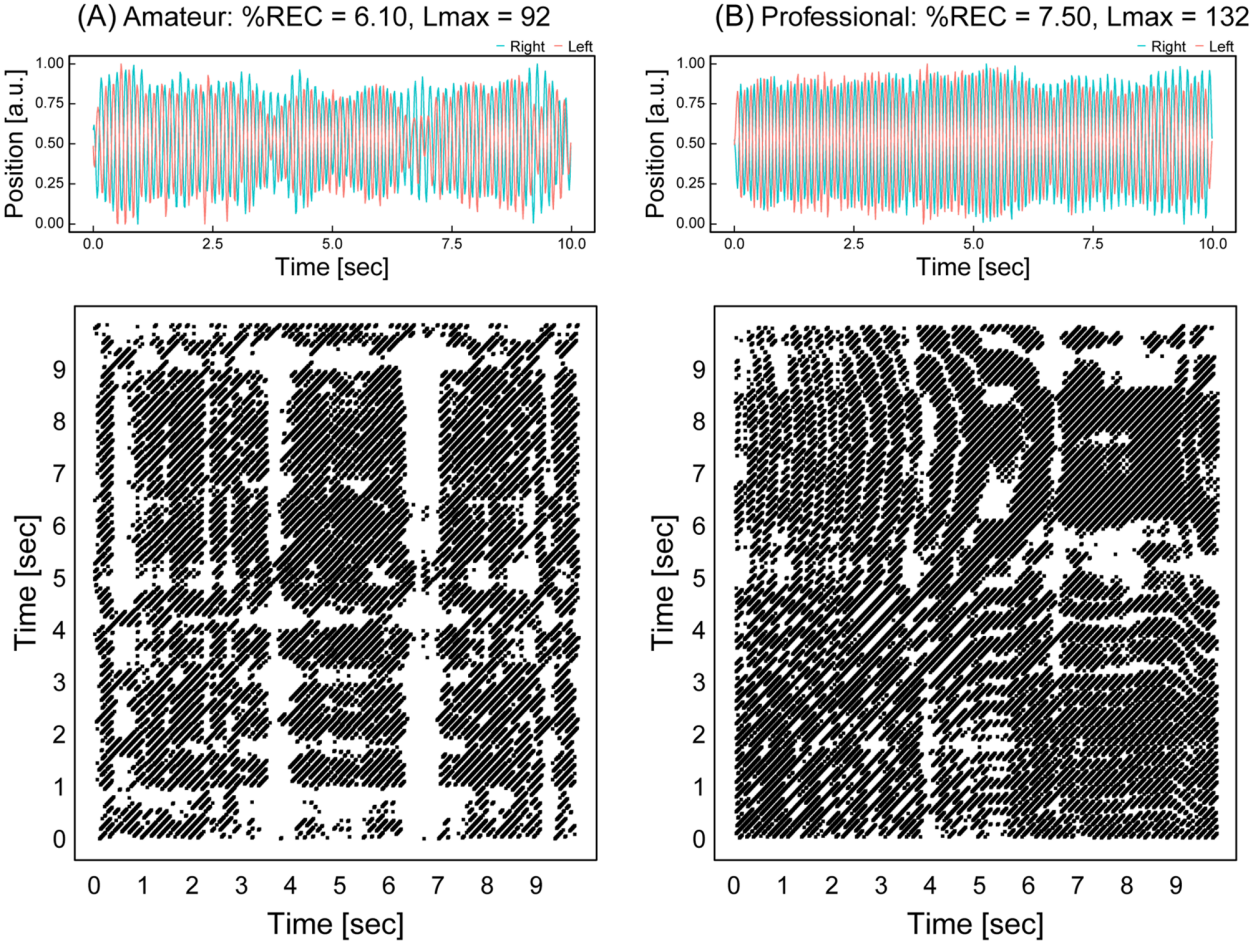
Typical examples of left- and right-hand time-series data (upper panels) and recurrence plots (lower panels) of an amateur player **A** and a professional player **B**

**Fig. 5:**
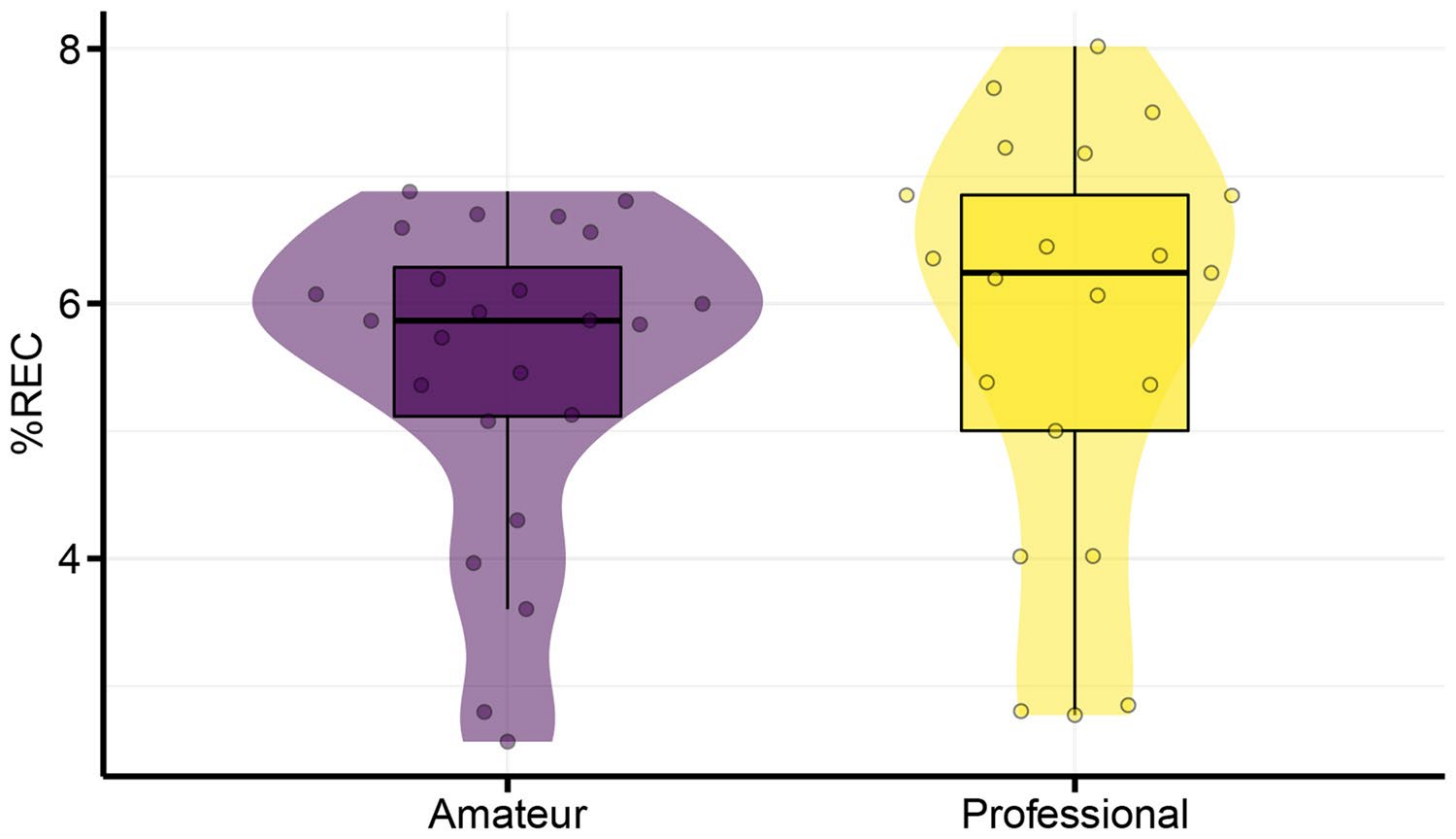
%REC of amateur and professional darbuka players. Each dot represents an observation for a participant’s trial

Figure 4 shows typical examples of left- and right-hand time-series data and recurrence plots of an amateur player and a professional player. Figure 5 shows the %REC of amateur and professional darbuka players. The LMM on the %REC showed that the main effect of group was not significant (${\chi }^{2}$(2) = 0.22, *p* = 0.64). The professional players showed less noise, but the difference was not significant (*β* = 0.27, *t* = 0.46, *95% CI*  − 0.86–1.40).

**Fig. 6:**
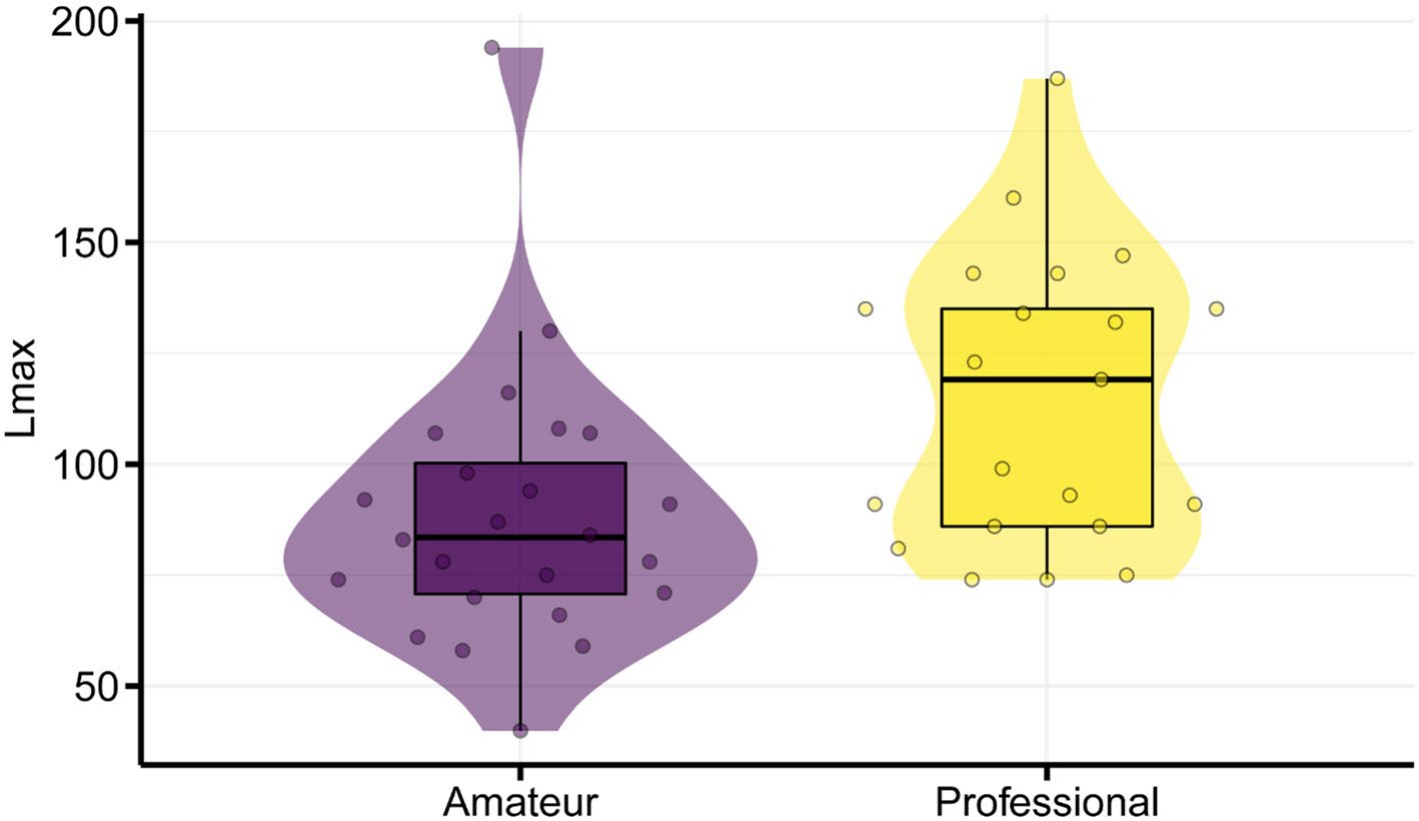
Lmax of amateur and professional darbuka players. Each dot represents an observation for a participant’s trial

Figure 6 shows professional and amateur players’ Lmax for bimanual tapping. The LMM on the Lmax of fast bimanual tapping showed that the main effect of group was significant (${\chi }^{2}$(2) = 5.15., *p* = 0.023, ${\eta }_{p}^{2}$ = 0.28). Professional players showed a larger attractor strength (*β* = 26.29, *t* = 2.27, *95% CI* = 3.71–48.87). The blocking cross-validation results for the Lmax data showed that Model 2 (intercept and group) won in 1939 of the 2000 cases and returned a lower prediction error than Model 1 (intercept only).

## Discussion

This study investigated how stochastic noise and attractor strength differ between professionals’ and amateurs’ fast bimanual coordinated tapping. Motion data were obtained from a 12 s video of fast bimanual tapping and examined using relative phase analysis and CRQA. Relative phase analysis showed less variability in fast finger tapping by professional players than by amateurs. Consistent with related studies of drummers or pianists (Yamanishi et al. 1980; Fujii et al. 2010), we first confirmed a more stable bimanual coordination in professional darbuka players. CRQA results also showed that, although professionals had significantly greater attractor strength, they did not show a significantly lower noise magnitude. In other words, professionals showed less variability in bimanual coordination, suggesting great underlying attractor strength.

### Noise and stability in bimanual coordination

Stochastic noise processes disturb stability in bimanual coordination, as indicated by $Q$ in Eq. 1 (Schöner et al. 1986). In the context of motor learning and noise, signal-dependent noise increases as the number of motor commands increases. Jäncke et al. (2000) reported that M1 activity decreases during bimanual coordination in professional musicians; M1 activity during the fastest tapping decreases with training, suggesting an increase in motor commands (Koeneke et al. 2006). Thus, noise magnitude may be lower in professional musicians engaged in prolonged practice. However, our findings showed no significant main effect of %REC, a noise magnitude index. It is possible that the amount of noise did not differ between amateurs and professionals; simple noise-reduction concepts, such as those reported in previous studies may not explain bimanual coordination during fast movements.

### Attractor and stability in bimanual coordination

Professional darbuka players showed significantly larger Lmax, an attractor strength index. Based on Eq. 1, two factors can change attractor strength: the detuning parameter ($\Delta \omega$) and coupling strength ($a$, $b$). First, detuning parameter ($\Delta \omega$) could be smaller in professional darbuka players than in amateur darbuka players. The larger the detuning parameter, the larger the movement variability and the more unstable the dynamical system structure becomes. Related studies have shown that asymmetry between the left and right sides is smaller in musicians than in non-musicians (Jäncke et al. 1997; Fujii et al. 2010). Fujii et al. (2010) showed that $\Delta \omega$ associated with $SD\phi$ during the fastest drumstick tapping, and that professional drummers performed with less asymmetry. Based on the results of an exploratory analysis of the unimanual tapping data from our previous study, the means of $\Delta \omega$ in amateur and professional players were 0.85 (*95% *CI 0.40–1.30) and 0.45 (*95% *CI 0.074–0.77). These values of $\Delta \omega$ were obtained by calculating the difference in frequencies between the left and right ring finger-tapping tasks, using the sound data shown in our previous study (Honda and Fujii 2022). Although it should be noted that the task and measurement environment were different, the means of $\Delta \omega$ in non-drummer and professional drummer were 1.31and 0.05 in the study by Fujii et al. (2010). Furthermore, we calculated the ratio of upper/lower triangles of the recurrence plot and the diagonal-wise recurrence profile based on lag analysis to quantify the leader–follower relationship of the hands (Coco and Dale 2014). The means of the degree of asymmetry in amateur and professional players were 1.01 (*95% *CI 0.91–1.10) and 0.81 (*95% *CI  0.68–0.95), and the means of maximum lag of the recurrence rate in amateur and professional players were 0.03 (95% CI  − 0.14–0.20) and 0.08 (95% CI  − 0.09–0.23) (see Figs. S3, S4). These findings suggest that the difference in the degree of hand-skill asymmetry between professional and amateur darbuka players might be relatively smaller than the results by Fujii et al. (2010).

Second, coupling strength ($a$, $b$) at anti-phase could be stronger in professional darbuka players than in amateur darbuka players. The larger the coupling strength, the smaller the movement variability and the more stable the dynamical system structure becomes. In the previous study by Kelso (1984), it was shown that bimanual coordination becomes unstable as finger swing movement frequency increases because the coupling strength weakened during fast movement. In contrast, the tapping speed was faster in the professional than in the amateur darbuka players (Honda and Fujii 2022), showing that the professional darbuka players rather achieve more stable performance even at the faster speed. This is consistent with the previous studies that showed more stable bimanual coordination in musicians than in non-musicians (Yamanishi et al. 1980; Verheul and Geuze 2004). The intrinsic dynamics of musicians were characterized by stronger attractors than the intrinsic dynamics of non-musicians (Verheul and Geuze 2004), suggesting that the larger coupling strength ($a$, $b$) in Eq. 1 in the professional players than in the amateur players. It is possible that CRQA captured the larger coupling strength ($a$, $b$) in the professional musicians.

### Relationship between attractor and skill

What does it mean that only the attractor is strengthened in professional musicians who perform with less variability? The notion that strong attractors suggest either rigid or robust performance is open to debate. Lmax (attractor strength) is assumed to be a measure of the system’s sensitivity to perturbation (Pellecchia et al. 2005), and this can indicate a rigid or strong coupling of the system against changes or perturbations. Thus, strong coupling (great attractor strength) can be interpreted as rigidity rather than robustness or flexibility. On the other hand, professional musicians must perform in various external environments, such as improvisations or ensembles with different members. In addition, they must operate under different internal conditions such as various neural and metabolic states. The internal conditions during musical performance also could potentially include emotional and affective factors, such as anxiety and nervousness (e.g., Yoshie et al. 2009). Considering these situations, which professional musicians encounter, the strong attractors may also indicate robustness or flexibility. Future studies would be interesting to determine whether strong attractors suggest robust and/or rigid performance using fractal analysis (e.g., Nonaka and Bril 2014) or perturbation experimental paradigms (e.g., Richardson et al. 2007).

### Supplementary Information

Below is the link to the electronic supplementary material.

Supplementary file1 (PDF 540 KB)

## Data Availability

The datasets generated during and/or analyzed during the current study are available from the corresponding author on reasonable request.
