# Supplementary material for: Bimanual finger coordination in professional and amateur darbuka players
Source: Exp Brain Res. 2023 Sep 26;241(11-12):2645–54. doi: 10.1007/s00221-023-06703-9 (PMC10635936; doi:10.1007/s00221-023-06703-9)
Supplement: Supplementary file 1 — Supplementary file1 (PDF 540 KB) [file 221_2023_6703_MOESM1_ESM.pdf]

Supplementary Materials

Article title: Bimanual finger coordination in professional and amateur darbuka players

Journal name: Experimental Brain Research

Author names: Kazuaki Honda[1, 2] and Shinya Fujii[3]

Affiliations and e-mail address of the corresponding author:

[1] Graduate School of Media and Governance, Keio University, 5322 Endo, Fujisawa, Kanagawa, 252-0882, Japan

[2] NTT Communication Science Laboratories, NTT Corporation, 3-1 Morinosato Wakamiya, Atsugi, Kanagawa, 243-0124, Japan

[3] Faculty of Environment and Information Studies, Keio University, 5322 Endo, Fujisawa, Kanagawa, 252-0882, Japan

e-mail: kazuaki.honda.ku@hco.ntt.co.jp

Average mutual information (AMI)

We calculated average mutual information (AMI) for determining the optimal value of time delay, as highlighted in the work of Wallot & Mønster (2018). The AMI was calculated for the left and right limb trajectories across all participants and trials. We then found that a time delay value of 2 was observed in 62% of the instances (mean: 2.64, SD: 1.67, median: 2, range: 2-7, as illustrated in Fig. S1). This result was consistent with our choice of using a value of 2 for the time delay.

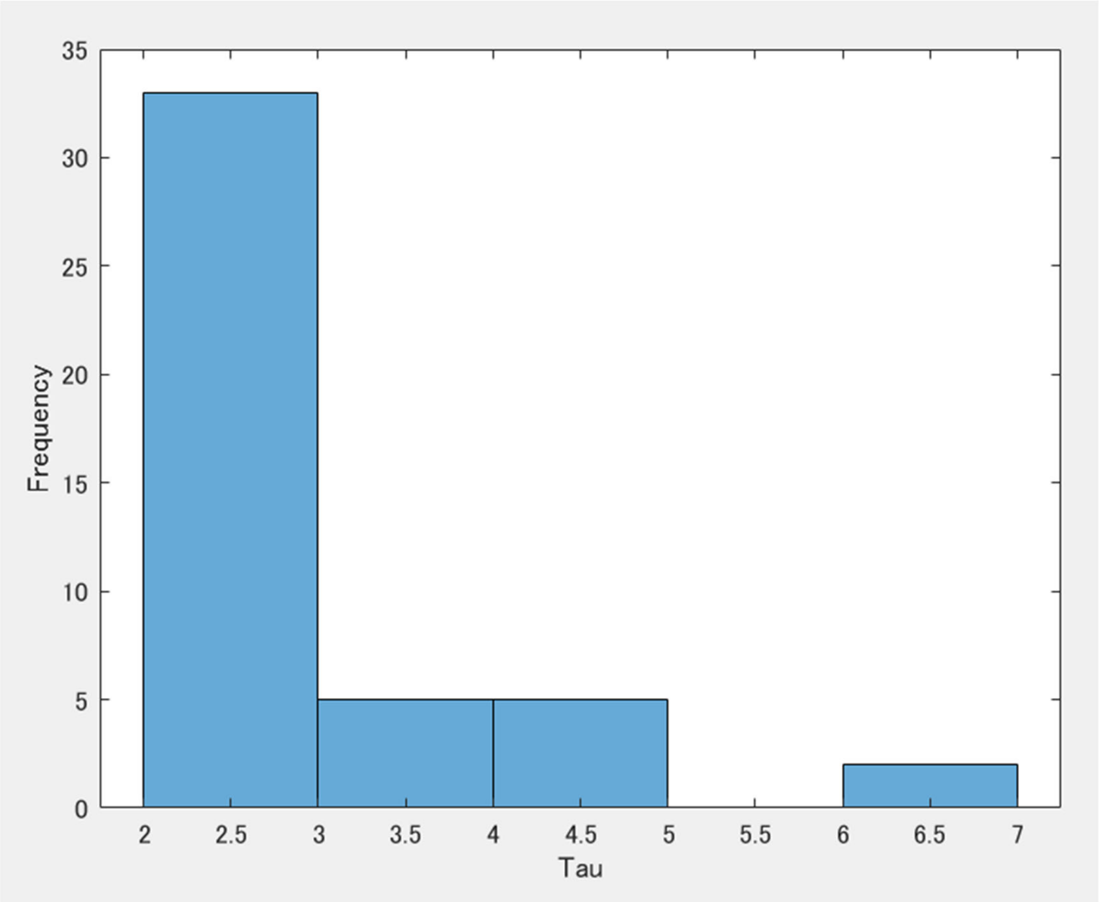

Fig. S1 Histogram of calculated τ for all observations.

False-nearest neighbors (FNN)

The previous studies by Goodman et al. (2000) and Mitra et al. (1997) have shown that an embedding dimension of five is well-suited for analyzing rhythmic limb movements (as further cited by Richardson et al., 2007). This notion from the previous studies was consistent with the FNN results above. Thus, we used the embedding dimension of five for our study. As a note, there is another criterion for %FNN (false-nearest neighbors) such as below 5% (Heggeret et al., 1999). When we calculated the FNN using the methods by Wallot & Mønster (2018), the results showed the embedding parameter of two or three dimensions can be adequate in satisfying the criterion below 5% (see Fig. S2).

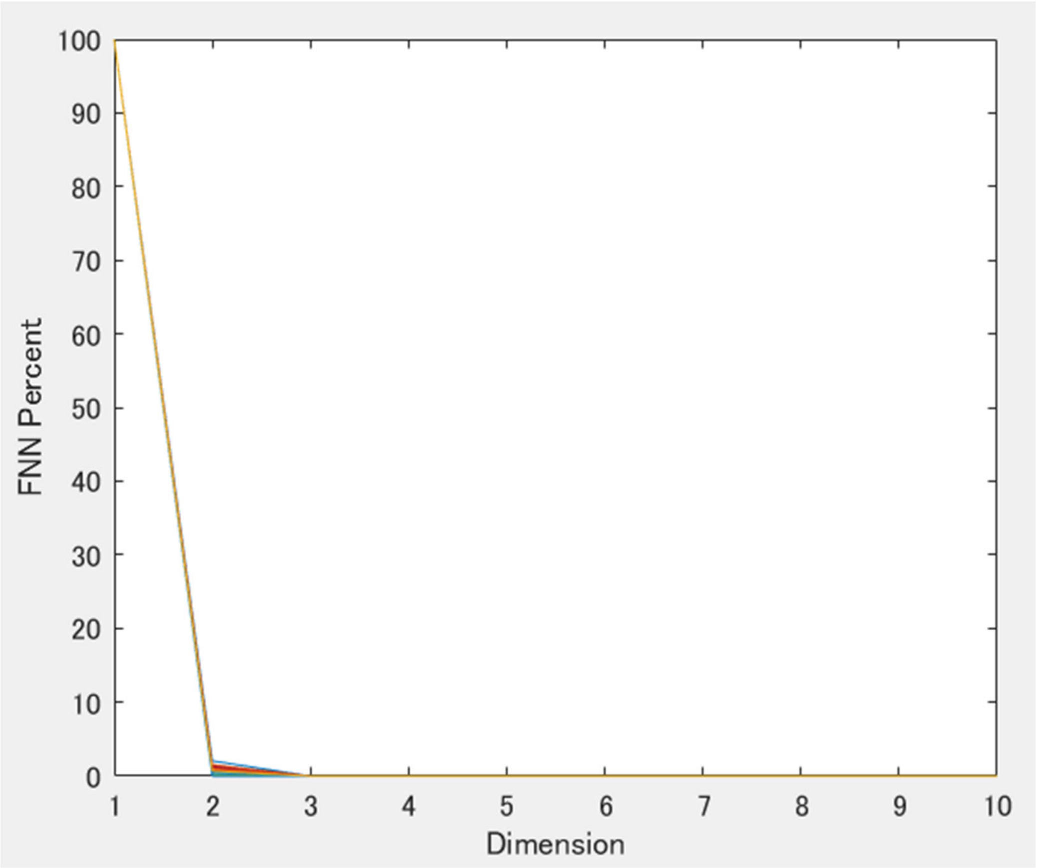

Fig. S2 FNN percentages for all observations.

The degree of asymmetry

We calculated the degree of the asymmetry of the ratio of upper/lower triangles of the recurrence plot. To calculate the degree of the asymmetry, we set the parameter "side" to "upper" or "lower" and calculated the ratio of the recurrence rate between "upper" and "lower". The means of the degree of asymmetry in amateur and professional players were 1.01 (*95% CI* = 0.91-1.10) and 0.81 (*95% CI* = 0.68-0.95).

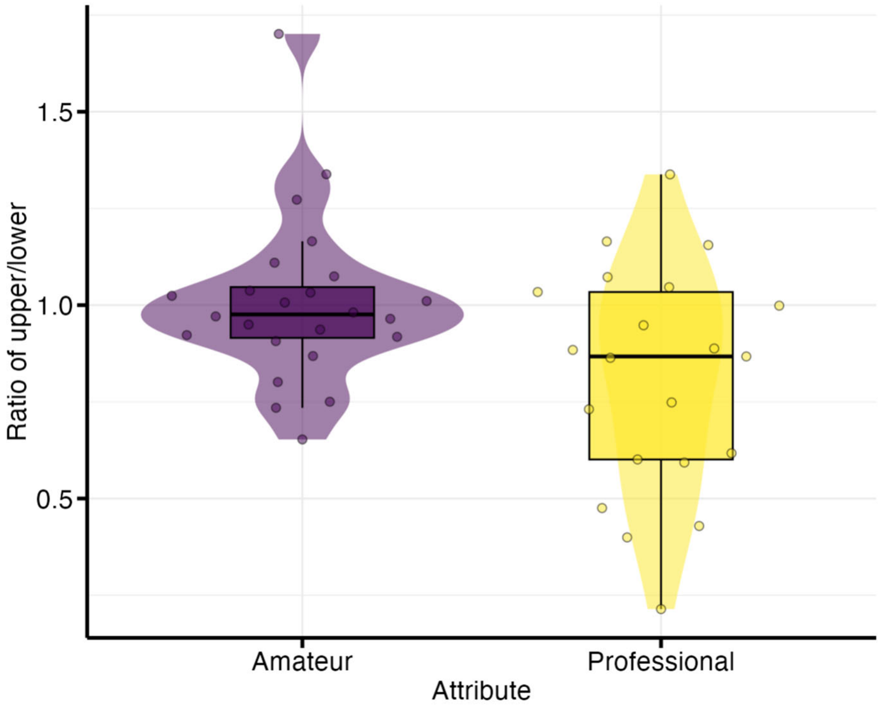

Fig. S3 The degree of asymmetry by calculating the ratio of upper/lower triangles of the recurrence plot.

The diagonal-wise recurrence profile

We calculated the diagonal-wise recurrence profile with a code by Coco and Dale (2014). The means of maximum lag of the recurrence rate in amateur and professional players were 0.03 (*95% CI* = -0.14 – 0.20) and 0.08 (*95% CI* = -0.09 - 0.23).

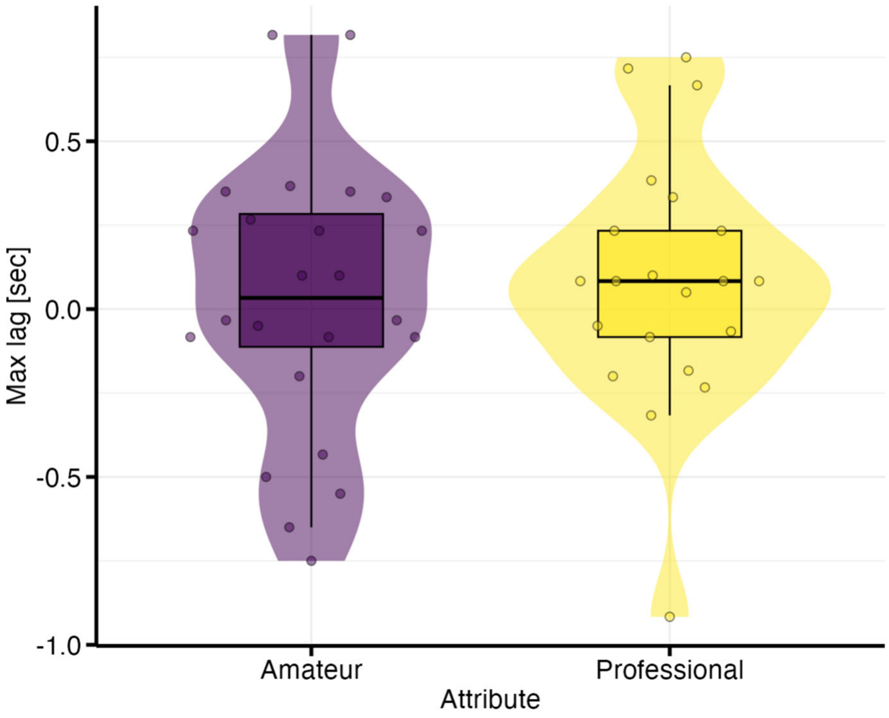

Fig. S4 The diagonal-wise recurrence profile based on lag analysis.

References:

Coco MI, Dale R (2014) Cross-recurrence quantification analysis of categorical and continuous time series: an R package. Front Psychol 5:510. https://doi.org/10.3389/fpsyg.2014.00510

Goodman L, Riley MA, Mitra S, Turvey MT (2000) Advantages of rhythmic movements at resonance: minimal active degrees of freedom, minimal noise, and maximal predictability. J Mot Behav 32:3–8. https://doi.org/10.1080/00222890009601354

Hegger R, Kantz H, Schreiber T (1999) Practical implementation of nonlinear time series methods: The TISEAN package. Chaos 9:413–435. https://doi.org/10.1063/1.166424

Mitra S, Riley MA, Turvey MT (1997) Chaos in Human Rhythmic Movement. J Mot Behav 29:195–198. https://doi.org/10.1080/00222899709600834

Richardson MJ, Schmidt RC, Kay BA (2007) Distinguishing the noise and attractor strength of coordinated limb movements using recurrence analysis. Biol Cybern 96:59–78. https://doi.org/10.1007/s00422-006-0104-6

Wallot S, Mønster D (2018) Calculation of Average Mutual Information (AMI) and False-Nearest Neighbors (FNN) for the Estimation of Embedding Parameters of Multidimensional Time Series in Matlab. Front Psychol 9:1679. https://doi.org/10.3389/fpsyg.2018.01679
